# Mitochondrial Reactive Oxygen Species: A Unifying Mechanism in Long COVID and Spike Protein-Associated Injury: A Narrative Review

**DOI:** 10.3390/biom15091339

**Published:** 2025-09-18

**Authors:** Eunseuk Lee, Adaobi Amelia Ozigbo, Joseph Varon, Mathew Halma, Madison Laezzo, Song Peng Ang, Jose Iglesias

**Affiliations:** 1 Department of Internal Medicine, Rutgers Health/Community Medical Center, Toms River, NJ 08755, USA; esesok@gmail.com (E.L.); songpengang@gmail.com (S.P.A.); 2 College of Integrated Health Sciences, University at Albany SUNY, Albany, NY 12222, USA; dobismelia@gmail.com; 3 College of Medicine, University of Houston, Houston, TX 77004, USA; josephvaron@yahoo.com; 4 Independent Medical Alliance, Washington, DC 20036, USA; mhalma@theflccc.org; 5 Open Source Medicine OÜ, Pärnu mnt. 139c, 11317 Tallinn, Estonia; 6 Department of Internal Medicine Hackensack Meridian School of Medicine Nutley, NJ 07110, USA; madison.laezzo@hmhn.org; 7 Department of Medicine, Division of Cardiology, Sarver Heart Center, University of Arizona College of Medicine, Tucson, AZ 85724, USA

**Keywords:** mitochondrial dysfunction, mitochondrial reactive oxygen species, SARS-CoV-2, long COVID, mitophagy, autophagy, biomarkers

## Abstract

Post-acute sequelae of SARS-CoV-2 infection (long COVID) present with persistent fatigue, cognitive impairment, and autonomic and multisystem dysfunctions that often go unnoticed by standard diagnostic tests. Increasing evidence suggests that mitochondrial dysfunction and oxidative stress are central drivers of these post-viral sequelae. Viral infections, particularly SARS-CoV-2, disrupt mitochondrial bioenergetics by altering membrane integrity, increasing mitochondrial reactive oxygen species (mtROS), and impairing mitophagy, leading to sustained immune activation and metabolic imbalance. This review synthesizes an understanding of how mitochondrial redox signaling and impaired clearance of damaged mitochondria contribute to chronic inflammation and multisystem organ symptoms in both long COVID and post-vaccine injury. We discuss translational biomarkers and non-invasive techniques, exploring therapeutic strategies that include pharmacological, non-pharmacological, and nutritional approaches, as well as imaging modalities aimed at assessing and restoring mitochondrial health. Recognizing long COVID as a mitochondrial disorder that stems from redox imbalance will open new options for personalized treatment and management guided by biomarkers. Future clinical trials are essential to validate these approaches and translate mitochondrial resuscitation into effective care for patients suffering from long COVID and related post-viral syndromes.

## 1. Introduction

Many viral infections, such as SARS-CoV-2, Cytomegalovirus, Influenza, among others, can result in post-infectious sequelae such as fatigue, exercise intolerance, brain fog, and dysautonomia [1,2]. Often these sequelae are precipitated by alteration of mitochondrial dynamics and, in part, mediated by reactive oxygen species, which persist after the end of the acute infection [3,4]. For viruses to replicate and propagate infection, they require the host cellular machinery to do so. The mitochondria are a critical organelle hijacked by viruses to reprogram the cell for viral replication [5]. Viruses alter mitochondrial bioenergetics and dynamics by altering mitochondrial membrane structure and function, mitochondrial electron transport, and other intermediaries [6]. These processes reprogram mitochondrial ATP production and beta oxidation of fatty acids, with the net effect being a redox state that maximizes viral assembly and replication. Additionally, virally induced damage alters the mitochondrial membrane potential, leading to an increase in mitochondrial reactive oxygen species (mROS). Viruses exploit mtROS to enhance viral replication [7].

Despite virally mediated cellular injury, it is beneficial for viral survival to prevent cellular programmed cell death (apoptosis). Viral infection disrupts the mitochondrial electron transport chain, leading to changes in the mitochondrial membrane potential, resulting in increases in mtROS, which activate mechanisms preventing apoptosis and activating mitophagy [8]. mtROS are essential in the initiation of autophagy and mitophagy, functioning as second messengers that trigger the AMP-activated protein kinase Unc-51 Like Autophagy Activating Kinase 1 pathway (AMPK-ULK-1) and stabilize the PTEN-induced putative kinase-1/Parkin RBR E3 ubiquitin protein ligase (Pink1–Parkin-mediated mitophagy pathway) [9]. It is evident that although mitochondria are the metabolic energy powerhouses of the cell and play a pivotal role in the regulation of the tricarboxylic acid cycle (TCA), fatty acid metabolism, oxidative phosphorylation, and the production of ATP, they are also vital constituents of the innate immune response [10].

Mitochondrial reactive oxygen species (mtROS) such as superoxide anion, hydrogen peroxide, hydroxyl radical, and nitric oxide are byproducts of oxidative phosphorylation [11]. Cellular respiration occurring in the mitochondrial electron transport chain is the major producer of mtROS. mtROS serve as second messengers in cell signaling, including apoptosis, mitophagy, autophagy, and other cell survival and cell death mechanisms [12,13,14,15]. They are essential in the proper functioning of the innate immune response [16]. mtROS are critical for orchestrating the innate immunological synapse, which is crucial in the organization of the innate immune response, the first line of defense during acute viral infection [16]. Although mtROS are essential to the organization of host responses in the innate immune system during acute viral infection, they can also become dysregulated, leading to post-viral chronic inflammatory states and other post-viral sequelae [17].

The recent COVID-19 pandemic demonstrates that many patients have suffered long-lasting and continued post-viral sequelae such as fatigue, exercise intolerance, dizziness, brain fog, depression, myocarditis, and dysautonomia [18]. It is pertinent to note that post-infectious sequelae are not exclusive to DNA and RNA viruses and have been known to occur in bacterial infections, such as Lyme disease and Q fever [19,20]. Viral infections altering mitochondrial dynamics, potentially leading to post-infectious sequelae, are summarized in Table 1.

Similarly, some patients have also suffered vaccine-mediated injury, not unlike post-viral syndromes [21,22]. This review will primarily focus on the mechanisms and potential treatment avenues for patients suffering from long COVID. To develop a working paradigm in the management of long COVID and vaccine-associated injury, it is essential to create an understanding of the interplay in viral manipulation of mitochondrial dynamics and the host’s immunologic and bioenergetic response. Mitochondria are not only the bioenergetic hub of the cell but also serve as an essential platform and regulator of innate immunity. Their injury during viral infection and convalescence represents a key driver in the pathogenesis of post-viral sequelae. Persistent virally induced dysregulation of mitochondrial bioenergetics leads to imbalances in redox states and excessive production of reactive oxygen species, which fuels a cascade of cellular events that precipitate chronic symptoms such as fatigue, exercise intolerance, brain fog, and autonomic dysfunction. Therefore, this review will discuss the role of mtROS in innate immunity, their role in the pathophysiology of long COVID, and post-vaccine injury. We also explore mitochondrial resuscitation, ranging from antioxidant therapies to metabolic modulators, which may provide valuable avenues in restoring cellular homeostasis and mitigating the effects of long COVID and post-vaccine injury.

**Table 1:** Viral Infections and Mitochondrial Dysfunction Potentially Leading to Post-Viral Sequelae.

Viral Infection	Mitochondrial Alteration	Mechanism/Effect	Functional Consequence	Reference
HIV	Enhanced fission; loss of ΔΨm; mtDNA depletion; reduced ATP-linked respiration	DRP1 upregulation; interaction with mPTP; ETC impairment	Mitochondrial fragmentation, apoptosis, and reduced energy production; neuropathy/myopathy	[20,23]
HCV	Enhanced fission; disruption of Ca^2+^ homeostasis; impaired ETC (complex I inhibition); mtDNA damage/depletion	DRP-1 increase; altered Ca^2+^ signaling; ROS generation	Mitochondrial fragmentation, oxidative stress, shift to glycolysis (Warburg effect), energy imbalance, and HIF-1α stabilization; depression, neurobehavioral dysfunction	[20,23]
HBV	Enhanced fission; disruption of Ca^2+^ homeostasis; loss of ΔΨm; mtDNA deletion/depletion	DRP-1 upregulation; VDAC/ANT interactions; apoptosis induction	Mitochondrial injury, apoptosis, progression of fibrosis/cirrhosis, fatigue, depression	[20,23]
EBV	Enhanced fission	DRP-1 activation	Increased mitochondrial fragmentation, linked to oncogenesis	[23]
SARS-CoV	Induces fusion; degradation of fission molecules	Degradation of DRP1 by ORF9b, mitochondrial fusion/elongation, and suppression of MAVS signaling	A more stable mitochondrial network supporting viral replication increases ROS; similar post-viral sequelae of SARS-CoV-2	[24]
SARS-CoV-2	Fusion, inefficient bioenergetics, increased mROS, mtDNA reduction (circulating and cellular), and immune evasion	ORF9b fusion induction and fission molecule degradation; OXPHOS inhibition → mROS → HIF-1α stabilization; ORF10-mediated MAVS inhibition; depletion of mtDNA in microglia and blood cells	Increase in glycolysis to fuel replication; chronic persistence/long COVID reservoirs; reduced mtDNA linked to higher mortality; attenuated IFN hyperinflammation via DAMP signaling; “long COVID”	[19,23]
Influenza Virus	Fusion and biogenesis; loss of ΔΨm; mtDNA release; MAVS inhibition	M2 → OPA1/MFN1-2 upregulation; PB1-F2 → ΔΨm dissipation, Cyt C release; M2 viroporin activity → mtDNA release	Increased mitochondrial number; apoptosis; immune suppression; activation of inflammasomes; cytokine storm. Fatigue, depression, encephalopathy	[20,23,25]
HSV-1	mtDNA depletion; Disruption of Ca^2+^ homeostasis; reduced ATP; ROS generation	UL12.5 causes mtDNA degradation; oxidative stress induction	Reduced respiration, impaired bioenergetics; depression	[20,23]
HTLV-1	Loss of ΔΨm; disruption of Ca^2+^ homeostasis	Alters inner membrane ion homeostasis	Induction of apoptosis via Cyt C release	[23]
CMV	Anti-apoptotic (prevents ΔΨm loss); Disruption of Ca^2+^ homeostasis; metabolic reprogramming	vMIA localizes to mitochondria; recruits BAX	Apoptosis inhibition to prolong infection; Warburg shift	[23]
HHV-8	Decreased mitochondrial biogenesis; suppressed OXPHOS; increased lactate production; disruption of Ca^2+^ homeostasis	Viral proteins (VGPCR, LANA, vCyclin, vFLIP) stabilize HIF-1a, upregulating glycolytic enzymes (PKM2, PDK1) and glucose transporters	Warburg shift → promotes survival, proliferation, and tumorigenesis of infected cells	[23,26]
HPV	Inhibition of apoptosis	Downregulates BAX-dependent pathways (via E6/E7)	Prevention of host cell death → persistence of infection	[23]
Encephalomyocarditis virus	mtDNA release	Viroporin 2B-mediated disturbance of mitochondrial membranes; MAVS-dependent translocation of mtDNA into the cytosol; activates NLRP3	mtDNA leakage into cytosol → immune activation and inflammation	[23,25]

**Abbreviations:** ΔΨm, mitochondrial membrane potential; ATP, adenosine triphosphate; BAX, Bcl-2-associated X protein; Ca^2+^, calcium; CMV, cytomegalovirus; Cyt C, cytochrome c; DRP1, dynamin-related protein 1; EBV, Epstein–Barr virus; ETC, electron transport chain; HBV, hepatitis B virus; HCV, hepatitis C virus; HHV-8, human herpesvirus-8; HIF-1α, hypoxia-inducible factor 1 alpha; HIV, human immunodeficiency virus; HPV, human papillomavirus; HTLV-1, human T-cell leukemia virus type 1; IFN, interferon; MAVS, mitochondrial antiviral signaling protein; mPTP, mitochondrial permeability transition pore; mROS, mitochondrial reactive oxygen species; mtDNA, mitochondrial DNA; NLRP3, NOD-, LRR- and pyrin domain-containing protein 3; OPA1, optic atrophy protein 1; OXPHOS, oxidative phosphorylation; PB1-F2, polymerase basic protein 1–frame 2 (influenza viral protein); PDK1, pyruvate dehydrogenase kinase 1; PKM2, pyruvate kinase M2; SARS-CoV, severe acute respiratory syndrome coronavirus; SARS-CoV-2, severe acute respiratory syndrome coronavirus 2; vFLIP, viral FLICE inhibitory protein; vGPCR, viral G protein-coupled receptor; vMIAs, viral mitochondria-localized inhibitors of apoptosis; VDAC, voltage-dependent anion channel.

## 2. Disambiguating Long COVID and Post-Acute COVID-19 Vaccination Syndrome

Long COVID and Post-acute COVID-19 vaccination syndrome (PACVS) both share a similar clinical phenotype [27] and, as such, may be difficult to disambiguate. Their similarity may stem from a similar pathology. Vaccination before COVID-19 infection has been shown in large-scale studies to reduce the risk of developing long COVID [28,29,30,31].

However, the response is more heterogeneous in cases of vaccination after long COVID. In most studies, administering a COVID-19 vaccine to someone with a case of long COVID produces, on average, symptom improvement [32,33,34]. Nevertheless, some studies show that some LC symptoms worsen on average after vaccination [35,36]. Additionally, in studies showing on-average improvement, a subset of patients (about 20%) deteriorate after vaccination [32,33], suggesting there may be variations in susceptibility [37]. One mediating factor that may explain the apparent contradiction is that people will only be diagnosed with long COVID, or believe they have it, unless there is a history of a previous infection. The relationship between SARS-CoV-2 infection and LC is not clear-cut, as the development of LC is similar between those with a confirmed prior infection and those without [38,39]. According to survey estimates, approximately 0.9% of those receiving a vaccine may develop Post-Acute COVID-19 Vaccination Syndrome (PACVS) [40], with a similar clinical presentation to LC [27,41]. Given the similar clinical presentation, it may be possible that some proportion of diagnosed LC cases are being conflated with PACVS. Given that there is little awareness of PACVS and hostility in some cases [22,42], a diagnosis of LC may be more readily made. Two-thirds of PACVS patients visited four or more doctors before receiving a diagnosis, suggesting rare acknowledgement [43].

In summary, the separation of LC and PACVS diagnoses may not be a straightforward task, especially considering the limited research available on PACVS and institutional reluctance to acknowledge and study it [40].

## 3. Sources and Functional Roles of ROS in Macrophages

Reactive oxygen species (ROS) are chemically reactive molecules derived from oxygen, encompassing both free radical and non-radical forms, including superoxide anion (O_2_^−^), hydrogen peroxide (H_2_O_2_), hydroxyl radical (OH^−^), ozone (O_3_), and singlet oxygen (O_2_) [44,45,46]. They are broadly derived from cytosolic and mitochondrial sources [47]. The cytosolic sources are the NADPH oxidase (NOX) family and xanthine oxidase. In contrast, mitochondria generate ROS through electron leakage from the electron transport chain (ETC), as well as from enzymes such as monoamine oxidases (MAO-A and MAO-B) and the adaptor protein p66Shc [47]. 

Among cytosolic sources, the NOX isoforms are the most characterized. In immune cells, NOX2 drives the oxidative burst during phagocytosis, and its deficiency explains the recurrent infections seen in chronic granulomatous disease. Beyond this immune role, NOX enzymes also participate in non-phagocytic processes [47]. NOX1 and NOX2 are involved in monocyte to macrophage differentiation and M2-type polarization, and NOX2-derived ROS have been implicated in the development of fatty liver disease [47]. In retinal phagocytes, NOX1 is the primary source of ROS and depends on the translocator protein (TSPO) for activation; this NOX1-TSPO activity in microglia has been linked to neurotoxicity and abnormal retinal angiogenesis in age-related macular degeneration in a mouse model [47,48]. Meanwhile, NOX4 can trigger macrophage death in response to oxidized low-density lipoprotein, tying cytosolic ROS production to the development of atherosclerosis [47,49].

Mitochondrial ROS (mtROS) serve as equally essential regulators, bridging cellular metabolism with innate immunity [47]. In phagocytes, electron leakage from the electron transport chain (ETC), particularly through reverse electron transport at complex I, amplifies inflammatory responses, such as LPS-induced cytokine production [50]. In addition, MAO-B-derived hydrogen peroxide (H_2_O_2_) supports NLRP3 inflammasome activation [51]. At the same time, mtROS contribute to non-phagocytic pathways: MAO-A activity influences catecholamine turnover in adipose tissue and sympathetic neuron-associated macrophages, thereby linking mtROS to systemic metabolic regulation [47]. The adaptor protein p66Shc further integrates oxidative stress with immune dysfunction by generating mitochondrial H_2_O_2_ and contributing to macrophage-derived foam cell formation in atherosclerotic lesions [44,47,52].

Together, both NOX-dependent cytosolic ROS and mtROS are indispensable to macrophage biology. They deliver rapid antimicrobial defense and shape immune signaling, but they also contribute to non-phagocytic processes, such as lipid metabolism, neurodegeneration, and vascular remodeling [47,53,54,55]. 

## 4. Mitochondrial ROS in Antiviral Innate Immunity

During viral infection, mitochondria act as signaling hubs, where mtROS generated from the ETC regulate innate immune defenses [4,56]. Following infection, the host’s innate immune system utilizes various pattern recognition receptors, including Toll-like receptors and retinoic acid-inducible gene-I (RIG-I)-like receptors (RLRs), to identify pathogen-associated molecular patterns (PAMPs) [57]. The RLRs, including RIG-I and MDA5, function as sensors within the cytoplasm, detecting the presence of viral RNA. Upon detecting a viral infection, these RLRs form a complex with their adaptor protein, the mitochondrial antiviral signaling protein (MAVS, also referred to as IPS-1, CARDIF, or VISA), which is located on the outer mitochondrial membrane and peroxisomes [58]. This complex is formed through interactions involving caspase activation and recruitment domains (CARDs). Subsequently, it recruits the IκB kinase (IKK) and TBK1/IKKi complexes, which trigger the expression of type I/III interferon. This then facilitates the translocation of transcription factors IRF3, IRF7, and IRF9, as well as NF-κB, thereby initiating the innate antiviral response [57,58]. To contain viral spread, MAVS interacts with several proteins, including DDX3, IKKi/IKKε, TRAF3, TRAF5, NEMO, WDR5, IRF3, IRF7, and STING. MAVS also interacts with proteins such as NLRC5, NLRX1, TRAF2, TRAF5, TRAF6, TAK1, and IKKα/β to mediate inflammatory responses [56].

Mitochondrial reactive oxygen species (mtROS) regulate antiviral signaling. They enhance MAVS activation and strengthen host defense when present in moderate concentrations. Transient oxidative surges can inhibit viral replication by facilitating MAVS oligomerization and enhancing the IRF3/NF-κB pathway, whereas an overly reduced redox environment (e.g., by excess antioxidants) impairs this signaling [47,56,58]. Consistent with this, pre-treatment of cells with antioxidants markedly reduces MAVS signaling, leading to a diminished interferon response and heightened viral load, highlighting the essential function of mtROS as a cofactor in antiviral immunity [4,58]. This link is particularly vital in mitochondrial health. Under typical conditions, mitophagy (a selective type of autophagy) eliminates impaired or damaged mitochondria to prevent excessive immunological activation. This process enables the cell to maintain metabolic integrity and prevent abnormal immunological signals [59]. When mitophagy is compromised, as observed in specific chronic infections or inflammatory conditions, defective mitochondria accumulate, hence exacerbating oxidative stress. Increases in oxidative stress consequently induce persistent MAVS activation and inflammasome engagement, primarily through NLRP3 and caspase-1, resulting in the maturation and secretion of IL-1β and additional pro-inflammatory cytokines [56]. This cascade is essential for acute antiviral defense; however, its sustained activation may lead to maladaptive responses, which can worsen immune dysfunction and contribute to persistent post-viral inflammation. Pathological states, such as SLE, highlight that excessive mtROS alone can drive MAVS oligomerization and IFN production in the absence of viral triggers [60].

## 5. Mitochondrial Stress and MAVS Dysregulation

Mitochondrial homeostasis reflects a continuous cycle of fusion, fission, biogenesis, and selective removal (mitophagy) [61]. Functionally competent (good) mitochondria maintain a stable membrane potential, support efficient ATP production, and regulate ROS levels appropriately, whereas dysfunctional (bad) mitochondria have impaired ATP production and are prone to excessive ROS generation and oxidative stress [62]. Fusion allows healthy mitochondria to compensate for localized stress. It is coordinated by MFN1/MFN2 (outer membrane) and OPA1 (inner membrane) and enables the exchange of material between partially damaged mitochondria to buffer local defects [63,64]. DRP1 drives fission and segregates damaged segments so they can be culled [63,65]. PINK-Parkin mitophagy then tags dysfunctional mitochondria for autophagic clearance, restraining mtROS, mitochondrial DNA (mtDNA) leakage, and downstream NLRP3 inflammasome activation [66,67]. Multiple studies show that intact mitophagy limits inflammasome activity and IL-1β maturation, whereas impaired mitophagy permits mtROS/mtDNA-driven NLRP3 signaling and excessive cytokine release [61,68,69]. 

Viruses systematically exploit the host’s mitochondrial fusion-fission mechanisms to subvert cellular homeostasis, culminating in excessive ROS production [70]. Viral proteins distort mitochondrial form and drive oxidative stress, targeting key fusion mediators (MFN1, MFN2, and OPA), and fission regulator (DRP1) [71]. For example, the dengue virus encodes a protease, NS2B3, that cleaves MFN1 and MFN2, thereby tipping the balance toward mitochondrial fragmentation [71]. Hepatitis C virus (HCV) also drives DRP1-dependent fission, and its NS5A protein triggers profound mitochondrial fragmentation, concurrently inhibiting electron transport chain complex I, which causes a loss of membrane potential and elevated ROS generation [70]. 

SARS-CoV-2 also provides a well-studied example. SARS-CoV-2 inhibits mitophagy by disrupting adapter proteins (it blocks p62-LC3 binding), causing damaged mitochondria to accumulate. Influenza A virus similarly targets autophagy; its M2 protein binds MAVS, elevates mtROS, and prevents MAVS clearance [4]. The result is a vicious cycle; mitochondria become fragmented and dysfunctional, mtROS remain high, and innate signaling (MAVS and inflammasomes) becomes dysregulated. SARS-CoV-2 RNA localizes to mitochondria in infected cells, and several viral proteins target mitochondrial components. The SARS-CoV-2 M protein binds complex I; NSP6 interacts with complex V; ORF10 interacts with inner membrane TIMM8; and ORF9b binds outer membrane TOMM70 [72]. These interactions disrupt mitochondrial function, causing swelling, membrane damage, and increased ROS production. Viral particles found near the mitochondrial matrix point to a direct viral attack on the structure and stability of mitochondria. Also, the virus blunts MAVS; SARS-CoV-2 ORF9b and NSP5 drive MAVS degradation via recruiting ubiquitin and breaking down RIG-I, respectively [72]. ORF10 translocates to mitochondria through interaction with the mitophagy receptor NIX, triggers LC3B recruitment, and selectively eliminates MAVS [73]. Additionally, the SARS-CoV-2 ORF3a protein has been implicated in the permeabilization and fragmentation of the mitochondrial membrane [70]. Together, these virus-induced distortions of homeostasis disrupt electron transport and promote the accumulation of dysfunctional mitochondria, fueling intracellular ROS accumulation [70].

Typically, the PINK1–Parkin pathway of mitophagy helps dampen prolonged MAVS signaling. PTEN-induced kinase 1 (PINK1), a mitochondrial kinase, promotes autophagic removal of damaged mitochondria and directly assists in degrading aggregated MAVS [74]. Recent studies show that PINK1 loss leads to MAVS overload [74,75]. PINK1-deficient cells accumulate MAVS multimers that persist after stimulation. These cells exhibit high IFN-β and IL-1β responses to RLR activation or stress, which are entirely dependent on MAVS [74]. Therefore, failure of PINK1-dependent mitophagy in chronic infection or stress can prevent timely MAVS clearance, fueling sustained cytokine release and pathology. When too much ROS builds up and mitophagy fails, it can set off a vicious cycle of MAVS and inflammasome activation that fuels excessive inflammation and ultimately causes tissue damage.

## 6. Mitochondrial Dysfunction and Redox Signaling in Long COVID 

### 6.1. Long COVID as a Mitochondrial Disorder

Long COVID, or post-acute sequelae of SARS-CoV-2 infection (PASC), continues to impact millions globally. Many patients experience persistent fatigue, brain fog, shortness of breath, or dysautonomia, sometimes for months after their initial infection [76]. These symptoms are often out of proportion to imaging or lab results, and frustratingly, many affected individuals appear "normal" on paper. Large epidemiologic studies have shown that this syndrome disproportionately affects women, racial minorities, and migrants, further highlighting disparities in both care and pathogenesis [77,78,79]. What is becoming clear is that we may be looking in the wrong places. Instead of lingering viral particles or structural damage, long COVID appears to be rooted in something more fundamental: a breakdown in cellular energy and immune balance, driven by mitochondrial dysfunction and oxidative stress. 

### 6.2. Viral Hijack and Metabolic Reprogramming

From the earliest days of infection, SARS-CoV-2 exploits the host’s mitochondria. Multiple viral proteins (ORF3a, ORF5, ORF6, ORF9c, ORF3c, ORF7b, ORF10, NSP4, NSP8, M, E proteins, and nucleocapsid) target distinct mitochondrial sites to destabilize homeostasis and blunt innate immunity and further impair respiration, induce mitophagy, elevate ROS, and promote immune evasion (see Table 2 for details) [73,74,75,76,77,78,79,80,81,82,83,84,85,86,87,88,89,90,91,92,93,94,95,96,97,98,99,100]. For example, ORF3a forms ion channels at the outer mitochondrial membrane to disturb calcium balance and trigger apoptosis, ORF5 downregulates MAVS-related signaling by limiting TBK1/IRF3 recruitment, while ORF6 alters the mitochondrial proteome while suppressing MAVS signaling [91,92]. ORF9b targets TOM70 on the outer mitochondrial membrane to silence MAVS signaling, blocking downstream IBK1/IRF3 activation and type I interferon production [93,94]. In contrast, E protein localizes mainly to ER/ERGIC/Golgi membranes, disrupting ER calcium stores and indirectly impairing ER–mitochondria calcium transfer, thereby contributing to mitochondrial dysfunction [80]. Together, these proteins drive excessive mitochondrial reactive oxygen species (mtROS) production [81,82,83,84,85,86,87,88,89]. Additionally, to increase mtROS in the acute phase of infection, calcium overload from ORF3a and E protein enhances reverse electron transport (RET) through Complex I, generating ROS bursts; in the chronic phase, proteins such as M (Complex I) and NSP6 (Complex V) inhibit the respiratory chain, sustaining electron leakage and oxidative stress [50,72] Together, these processes stabilize HIF-1α, enforce glycolytic reprogramming, and perpetuate inflammation. This increase in oxidative stress stabilizes hypoxia-inducible factor 1-alpha (HIF-1α), pushing cells away from oxidative phosphorylation (OXPHOS) and into glycolysis, a low-efficiency metabolic state more suited for acute stress and viral replication [90,93,94]. While this metabolic switch benefits the virus in the short term, it becomes maladaptive when sustained. The result is what many patients describe: a crushing lack of energy, poor stress tolerance, and brain fog that feels almost metabolic. This "glycolytic lock" mirrors what we see in other conditions like chronic fatigue syndrome or diabetic cardiomyopathy [19].

### 6.3. Immune Activation and the Vicious Cycle of Injury

**Figure 1:**
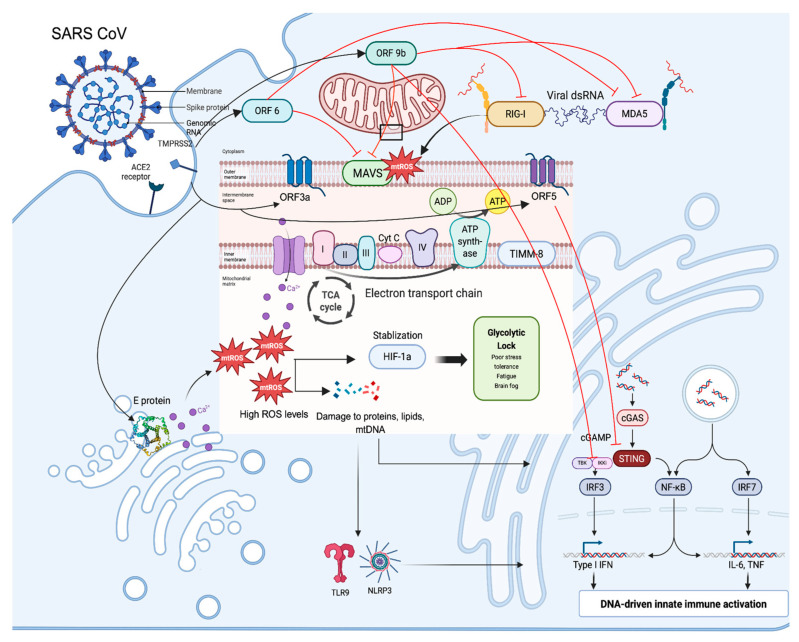
This diagram illustrates how SARS-CoV proteins (e.g., ORF3a, ORF5, ORF6, ORF9b, E protein) disrupt mitochondrial homeostasis. ORF3a localizes to the outer mitochondrial membrane via ER–mitochondria contact sites, where its calcium-permeable ion channel activity directly perturbs mitochondrial calcium balance, promotes mitochondrial fission, and triggers apoptosis and inflammasome activation. ORF5 downregulates MAVS MAVS-related signaling by limiting TBK1 and IRF3 recruitment. ORF6 interferes with MAVS and MDA5-TBK1-mediated interferon induction. ORF9b targets TOM70 on the outer mitochondrial membrane to suppress MAVS signaling, blocking downstream TBK1/IRF3 activation and type I interferon production. In contrast, the E protein primarily integrates into ER, ERGIC, and Golgi membranes, where its viroporin activity depletes ER calcium stores and indirectly impairs ER–mitochondria calcium transfer, thereby propagating mitochondrial dysfunction. Together, these proteins drive calcium dysregulation and excessive mitochondrial ROS (mtROS). Perturbed Ca^2+^ influx into mitochondria amplifies ROS generation and accelerates opening of mitochondrial permeability transition pore, further sensitizing cells to metabolic collapse. Elevated ROS stabilizes HIF-1α, shifting cell metabolism from oxidative phosphorylation (OXPHOS) to glycolysis, a state that favors acute viral replication but leads to persistent fatigue, stress intolerance, and cognitive dysfunction. ROS-induced damage to mtDNA leads to its leakage into the cytosol and bloodstream, where it is recognized by innate immune receptors such as cGAS–STING, TLR9, and the NLRP3 inflammasome. The activation of IL-6, IL-1β, and type I interferons contributes to long-term inflammation observed in long COVID patients. The diagram depicts a vicious cycle: mitochondrial damage → ROS → metabolic reprogramming → mtDNA leakage → immune activation → further mitochondrial injury.

**Figure 2:**
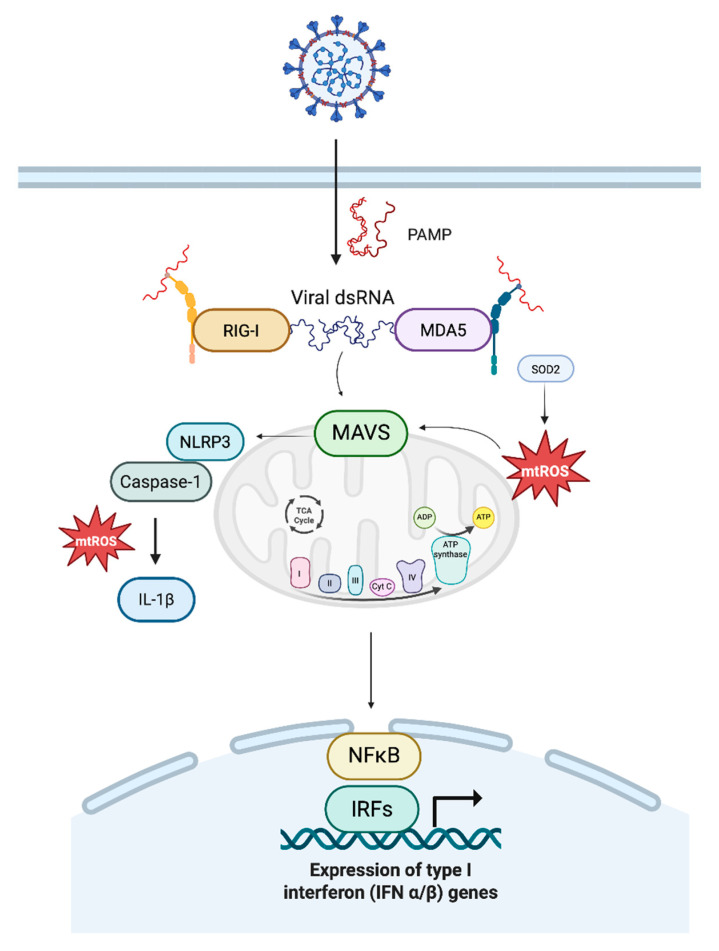
This diagram illustrates that viral infections introduce pathogen-associated molecular patterns (PAMPs) into the host cytoplasm, such as double-stranded RNA (dsRNA) and 5’ triphosphorylated single-stranded RNA, which are not typically present in host cells. These PAMPs are recognized by retinoic acid-inducible gene I-like receptors (RLRs), namely RIG-I and MDA5. Upon binding PAMPs, RIG-I and MDA5 undergo conformational changes that expose their caspase activation and recruitment domains (CARDs), allowing them to interact with mitochondrial antiviral-signaling protein (MAVS) on the outer mitochondrial membrane. This interaction promotes MAVS oligomerization, forming a platform for recruitment of downstream signaling molecules such as TBK1, IKKε, TRAF3, and NEMO, leading to phosphorylation and nuclear translocation of IRF3/IRF7 and activation of NF-κB. This cascade drives the production of type I and III interferons (IFN-α/β and IFN-λ) and pro-inflammatory cytokines, amplifying the innate immune response against viral replication. Importantly, mitochondrial reactive oxygen species (mtROS) and dysregulated Ca^2+^ influx both enhance MAVS signaling, with Ca^2+^ acting as a second messenger that amplifies IRF3/NF-κB activation, linking redox balance to antiviral defense. Failure to clear damaged mitochondria via mitophagy, particularly through the PINK1–Parkin pathway, results in persistent mtROS elevation and unrestrained MAVS signaling, potentially leading to chronic inflammation or autoimmunity.

mtROS not only impair energy production but also damage mitochondrial DNA (mtDNA), leading to its leakage into the cytoplasm and bloodstream. These fragments act as danger-associated patterns (DAMPs) or pathogen-associated molecular patterns (PAMPs), activating innate immune sensors such as cGAS–STING, Toll-like receptor 9 (TLR9), and the NLRP3 inflammasome [81]. Even after the virus is cleared, this immune activation persists, with many long COVID patients showing elevated levels of IL-6, IL-1β, and type I interferons weeks or months post-infection, thus creating a self-perpetuating loop: mitochondrial dysfunction leads to ROS, which causes metabolic reprogramming, mtDNA leakage, innate immune activation, and then further mitochondrial injury. Additionally, mtROS enhances signaling through the MAVS pathway, promoting oligomerization and amplification of antiviral responses via IRF3 and NF-κB. Inadequate clearance of damaged mitochondria, particularly via defective mitophagy through the PINK1–Parkin pathway, perpetuates chronic inflammation and immune dysregulation. (Figure 1 and Figure 2).

### 6.4. Organ System Impact and Clinical Correlates

Translational studies reveal mitochondrial dysfunction across multiple organ systems in long COVID. In the kidney, suppression of OXPHOS-related genes in peripheral blood mononuclear cells (PBMCs) during acute infection predicted worse renal outcomes a year later [107]. In the endocrine pancreas, infected β-cells exhibit mitochondrial fragmentation and accumulation of NADH, mimicking the metabolic features of type 2 diabetes and potentially explaining the rise in post-COVID diabetes [19,108]. In the cardiopulmonary system, transgenic mouse models treated with mitochondrial-targeted antioxidants such as EUK-8 and mCAT demonstrated restored OXPHOS, reduced inflammation, and improved survival [81]. These findings strongly support the view that mitochondrial distress is not just a bystander in long COVID; it is central to its pathophysiology. Endothelial dysfunction also appears linked to mitochondrial stress, as excess ROS disrupts nitric oxide signaling, impairing vascular tone and contributing to orthostatic intolerance and post-exertional malaise [109,110].

Additionally, mtROS excess has been implicated in endothelitis, microthrombosis, and myocardial fibrosis [111]. Although COVID-19 and mRNA-associated myocarditis are poorly understood and complex phenomena, altered mitochondrial dynamics and increases in mtROS may play a role in myocardial injury. Huynh et al. demonstrated that the S1 unit of spike protein in vitro impaired mitochondrial dynamics in human cardiomyocytes, altering mitochondrial membrane potential, resulting in increases in myocardial calcium and excessive ROS production [112]. Increases in mtROS accumulation activate NLRP inflammasome, resulting in increased myocardial fibrosis [113].

**Figure 3:**
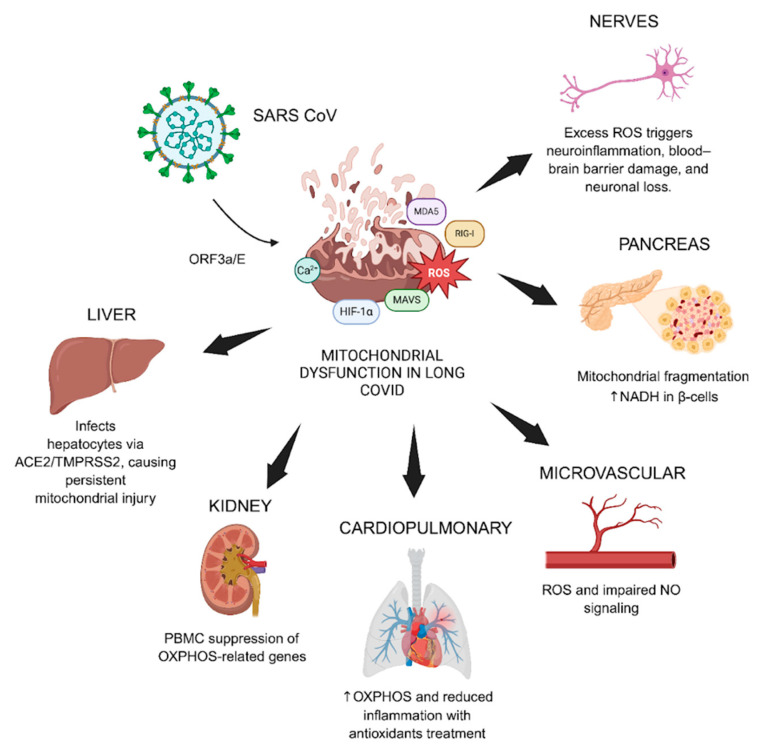
This diagram illustrates how mitochondrial dysfunction contributes to long COVID by affecting multiple organ systems. In the kidney, suppression of OXPHOS genes during acute COVID predicts worse outcomes months later. In the pancreas, infected β-cells show mitochondrial fragmentation and metabolic stress, similar to what is seen in type 2 diabetes. In the heart and lungs, antioxidant treatments that target mitochondria improve energy production, reduce inflammation, and improve survival in animal models. In the blood vessels, excess mitochondrial ROS impairs nitric oxide signaling, leading to poor circulation and symptoms like dizziness and post-exertional fatigue. Together, these findings show that mitochondrial damage plays a central role in long COVID symptoms.

In the liver, SARS-CoV-2 infects hepatocytes via ACE2 and TMPRSS2, including steatosis and persistent mitochondrial injury, even in those without prior liver disease [114]. This contributes to disease progression in patients with metabolic-associated steatohepatitis (MASH), especially in those with preexisting metabolic dysfunction. Persistent liver enzyme elevation, mitochondrial swelling, and altered redox signaling have been observed for up to 20 months post-infection, even in patients without prior liver disease [115,116,117]. Longitudinal studies showed elevated fibrosis indices and liver stiffness in long COVID cohorts, suggesting ongoing necroinflammation [114,118]. Parallel mechanisms appear in the nervous system [119,120]. Redox imbalance and immune dysregulation have been implicated in COVID-19-associated neuroinflammation, where mitochondrial dysfunction in neurons and glia fosters a pro-inflammatory state [120]. ROS-driven neuroinflammation and microglial activation, in part mediated by ACE2 depletion following viral entry, lead to mitochondrial injury. This injury is amplified by AngII/AT1R/Nox2-mediated ROS generation, compromising the blood–brain barrier and contributing to cognitive symptoms, anosmia, and neural apoptosis (Figure 3) [121,122].

The convergence of clinical, biochemical, and imaging data suggests that mitochondrial dysfunction is an upstream driver of persistent symptoms in long COVID. Redox-sensitive pathways regulate immune activation, vascular tone, and metabolic flexibility, all of which are commonly disrupted in post-COVID-19 syndromes.

The proposed mechanism, as synthesized across studies, involves acute viral injury triggering neutrophil activation and ROS production, followed by impaired resolution of oxidative stress in vulnerable individuals. This may be due to age-related reductions in endogenous antioxidant defenses, as noted by Laforge et al., who observed decreased SOD3 expression in elderly COVID-19 patients [123]. Continued ROS generation then perpetuates tissue injury, immune activation, and bioenergetic failure through a self-reinforcing cycle.

### 6.5. Biomarkers of Redox Imbalance and Mitochondrial Injury

Several biomarkers support the central role of mitochondrial dysfunction in long COVID. Studies have identified elevated levels of peroxiredoxin-3 (PRDX3), malondialdehyde, 8-hydroxy-2’-deoxyguanosine, and nitric oxide metabolites in symptomatic patients, alongside reduced antioxidant enzymes such as superoxide dismutase (SOD) and glutathione peroxidase (GPx) [19,124,125]. These findings suggest sustained redox imbalance that correlates with persistent symptoms such as fatigue, dyspnea, and brain fog. Transcriptomic profiling has shown upregulation of genes involved in ROS generation, impaired mitophagy, and mitochondrial fragmentation. Circulating mtDNA, a potent immune activator, correlates with cardiovascular complications and may serve as a dynamic marker for mitochondrial stress. Magnetic Resonance Spectroscopy (MRS) has emerged as a non-invasive tool to quantify oxidative phosphorylation capacity, revealing prolonged phosphocreatine recovery and reduced ATP synthesis in long COVID patients. These findings not only correlate with symptom severity but are also partially reversible with targeted nutritional interventions.

Shankar et al. conducted transcriptomic and proteomic profiling of individuals with long COVID and myalgic encephalomyelitis/chronic fatigue syndrome (ME/CFS), identifying convergent upregulation of genes related to ROS production, impaired mitophagy, and mitochondrial fragmentation [126]. These data support the hypothesis that mitochondrial redox dysfunction may be a shared driver of post-viral fatigue syndromes.

One of the most promising translational markers is mitochondrial DNA (mtDNA) damage. Semo et al. reported that individuals with PASC-related cardiovascular complications displayed evidence of sustained monocyte bioenergetic impairment and mtDNA fragmentation [127]. Circulating mtDNA acts as a damage-associated molecular pattern (DAMP), capable of activating innate immune sensors such as TLR9 and the cGAS–STING pathway, sustaining chronic inflammation long after viral clearance.

Magnetic resonance spectroscopy (MRS) has also emerged as a non-invasive technique to assess mitochondrial dysfunction. In a study by Chen et al., patients with post-COVID fatigue exhibited significantly delayed phosphocreatine recovery times (τPCr) and reduced maximal ATP synthetic capacity (Qmax) on skeletal muscle MRS, indicating impaired oxidative phosphorylation [128]. These MRS findings correlated with symptom severity and were partially reversible with personalized nutritional interventions.

Tsilingiris et al. reviewed the current literature on laboratory markers in long COVID. They highlighted alterations in multiple oxidative stress pathways, including reductions in reduced glutathione (GSH), increased lipid peroxidation, and elevated protein carbonylation [129]. These markers not only support the presence of mitochondrial dysfunction but may also serve as candidates for future diagnostic or prognostic algorithms.

Importantly, biomarkers such as MDA, d-ROMs, GSH/GSSG ratios, and mtDNA damage not only reflect disease severity but may be leveraged to stratify patients and monitor therapeutic response. Functional imaging modalities like MRS further enable real-time tracking of mitochondrial recovery and could be integrated into future clinical trials.

### 6.6. Therapeutic Interventions: Mitochondrial Resuscitation Targeting Mitochondrial Dysfunction

Therapies aimed at restoring mitochondrial function and redox balance are emerging as promising strategies. Mitochondria-targeted antioxidants such as MitoQ, EUK-8, and mCAT have shown preclinical benefits in reversing bioenergetic failure and reducing inflammation [19,81]. Over-the-counter agents such as coenzyme Q10 and α-lipoic acid may offer modest symptomatic relief by supporting mitochondrial respiration [130]. NAD⁺ precursors like nicotinamide riboside (NR) and nicotinamide mononucleotide (NMN) support mitochondrial biogenesis and DNA repair through sirtuin and PARP pathways and are currently being evaluated in clinical trials, including NCT05703074 [19]. N-acetylcysteine (NAC), a glutathione precursor, may reduce neutrophil-driven oxidative injury and mitigate endothelial damage [131,132]. Metformin, via AMPK activation and inhibition of mitochondrial complex I, suppresses ROS production and reduces systemic inflammation, offering potential benefit in patients with concurrent metabolic disorders [133,134]. Precision nutrition based on MRS and biomarker profiling, including supplements like creatine, magnesium, and riboflavin, has shown promise in restoring mitochondrial function and alleviating fatigue [135].

Targeting redox imbalance and mitochondrial injury presents a rational therapeutic strategy in long COVID. Several antioxidants and metabolic modulators have shown promise in observational studies and early clinical trials.

Coenzyme Q10 (CoQ10) is a mitochondrial electron carrier and lipid-phase antioxidant that has been studied in post-viral fatigue syndromes. A review by Mantle et al. found improvements in fatigue scores and mitochondrial function parameters in ME/CFS and post-viral fatigue patients following CoQ10 supplementation [136]. Given its central role in ATP generation and ROS neutralization, CoQ10 represents a potentially safe and effective intervention in long COVID.

N-acetylcysteine (NAC), a precursor to intracellular glutathione, has both antioxidant and anti-inflammatory properties. In the context of COVID-19, NAC has been proposed to reduce neutrophil-driven ROS production and limit oxidative tissue damage. Laforge et al. suggested that NAC, particularly when combined with neutrophil elastase inhibitors like sivelestat, could attenuate reverse-transendothelial migration (rTEM) of neutrophils and thereby reduce ROS-induced endothelial injury and thrombosis [123].

Metformin, while primarily used in diabetes management, has demonstrated the ability to reduce mitochondrial ROS production through AMPK activation and inhibition of mitochondrial complex I. In both ME/CFS and long COVID cohorts, metformin has been shown to suppress hyperactive T cell phenotypes and reduce systemic inflammation. Its metabolic effects may be particularly relevant in patients with concurrent metabolic syndrome or cardiovascular risk [137,138,139,140].

Nutraceutical approaches, including nicotinamide riboside (NR) and nicotinamide mononucleotide (NMN), support NAD⁺ biosynthesis, which is critical for mitochondrial sirtuin activation and DNA repair. These compounds have shown potential to reverse mitochondrial dysfunction in preclinical models and are currently under investigation in long COVID [141].

Precision nutrition, guided by biomarker and MRS profiling, may also play a role. Chen et al. demonstrated that patients with impaired muscle oxidative capacity on MRS benefited from tailored nutritional interventions targeting mitochondrial cofactors, including creatine, magnesium, and riboflavin [128]. These interventions improved both MRS parameters and subjective fatigue scores.

### 6.7. Exercise and Mitochondrial Rehabilitation

**Figure 4:**
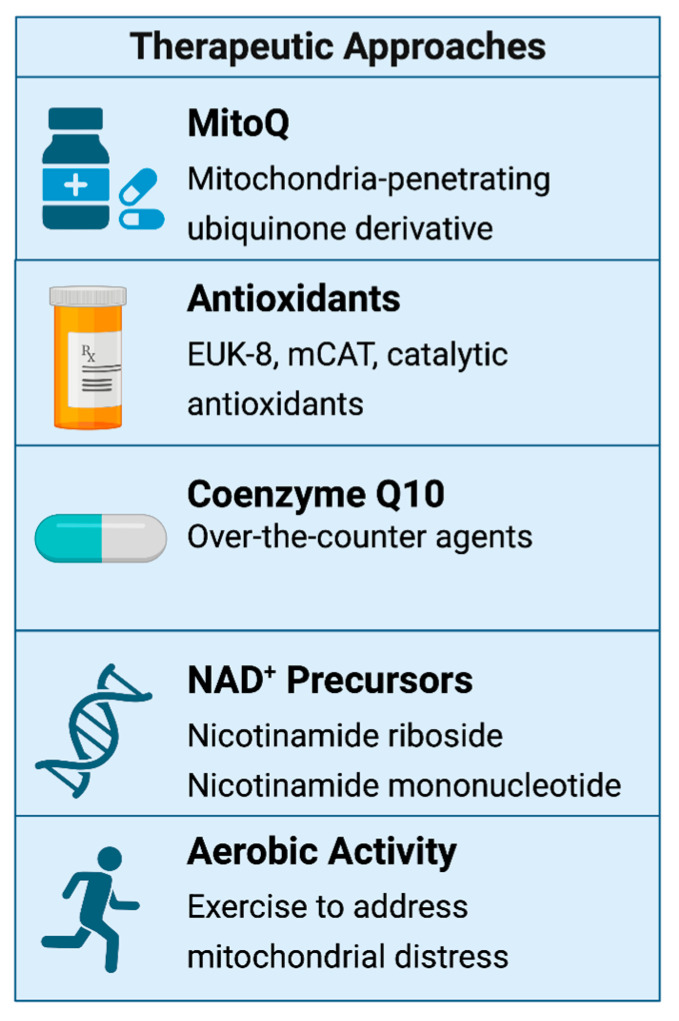
This diagram illustrates emerging treatments for long COVID that target mitochondrial dysfunction. It shows how therapies like MitoQ, EUK-8, mCAT, CoQ10, α-lipoic acid, and NAD⁺ precursors (NR, NMN) aim to restore mitochondrial function, reduce oxidative stress, and support energy production. It also highlights how light aerobic exercise, when timed appropriately, may help improve energy by boosting mitochondrial capacity. These strategies focus on treating the root cause, mitochondrial stress, rather than just managing symptoms.

Non-pharmacologic strategies such as light aerobic exercise, when timed appropriately, may also aid mitochondrial recovery. In one study, Nordic walking helped improve lactate handling and subjective energy levels, likely through enhanced mitochondrial capacity [142]. However, exertion must be carefully calibrated to avoid exacerbating symptoms during periods of active inflammation. This emphasizes the importance of identifying the right therapeutic window for rehabilitation, guided by clinical and metabolic markers. Therapeutic interventions are summarized in Table 3 and Figure 4. 

### 6.8. Translational Tools and Precision Medicine Outlook

The next step in translating this knowledge into clinical practice lies in identifying which patients have a mitochondrial-dominant phenotype. Emerging biomarkers may help. PBMC OXPHOS gene expression panels, such as those developed by Jayaraman and colleagues, could help stratify patients and monitor response [107]. Serum peroxiredoxin-3 (PRDX-3), a mitochondrial antioxidant enzyme, appears elevated in patients with long COVID-related fatigue and dizziness [19]. Circulating mtDNA levels, which correlate with mitochondrial stress and respond to antioxidant therapy, may serve as a dynamic, real-time marker for treatment efficacy [81]. These tools bring us closer to a precision medicine model where therapies are not just empiric but personalized. 

Dysautonomia and brain fog affect many individuals with long COVID, possibly due to neuroinflammation [151]. Given the pathophysiologic role of dysfunctional mitochondria and mtROS in long COVID and perhaps post-vaccine syndromes, from a translational medicine standpoint, it is of importance to revisit the repurposing of agents with overlapping anti-inflammatory and antioxidant properties such as Ivermectin, Dimethyl sulfoxide (DMSO), and Methylene blue (Mb). Either indirectly via anti-inflammatory mechanisms or through direct effects on mitochondrial electron transport chain, these agents have an impact on mitochondrial function pathways and generation of reactive oxygen species [152,153]. When discussing the protective role of antioxidants, it is essential to consider that, depending on the clinical scenario, the timing of administration, the doses administered, and the patient’s internal milieu, antioxidants can paradoxically induce oxidant stress [154,155].

The majority of evidence shows that ivermectin induces mitochondrial oxidant stress and increases reactive oxygen species [156,157]. The oxidant effect of ivermectin is not the case in all instances. For example, in a rat model of bleomycin-induced pulmonary fibrosis, Ivermectin resulted in a reduction in markers of oxidative stress [158,159]. Additionally, Ivermectin suppressed NLRP3, thus supporting the antioxidant and anti-inflammatory effects of Ivermectin [158]. In a rat model of methotrexate-induced liver injury, rats who received ivermectin demonstrated lower levels of malondialdehyde, presumably due to antioxidant and anti-inflammatory mechanisms [160]. In a rat model of transient cerebral ischemia, Seyyedabade et al. demonstrated that treatment with ivermectin resulted in a decrease in infarct size, lipid peroxidation, and myeloperoxidase [161]. De Melo et al. in a Syrian hamster model of SARS-CoV-2, Ivermectin prevented the development of anosmia and limited inflammation in the lungs of treated animals [162]. In a rat model of streptozotocin-induced Alzheimer’s disease, ivermectin attenuated amyloid plaque buildup by inhibiting acetylcholinesterase [163]. Thus, preclinical evidence supports the possibility that ivermectin may be an ideal candidate drug in reducing neuroinflammation in patients with long COVID.

Mb possesses a net positive charge and unique hydrophilic and lipophilic properties, enabling it to penetrate and accumulate in mitochondrial membranes [164,165]. Mb can function as an alternative electron transporter in the mitochondrial transport chain (MTC), receiving electrons from NADPH and can bypass complex I-III, transporting electrons directly to cytochrome C [166]. This mechanism may restore function to a damaged MTC and restore ATP production [166]. Additionally, Mb is a powerful redox agent and can function as a free radical scavenger and antioxidant [164]. Mb has also demonstrated in vitro virucidal activity against SARS-CoV-2 and H1N1 influenza viruses [167]. In a clinically relevant rat model of sepsis, Mb demonstrated a reduction in plasma levels of inflammatory cytokines IL-6 and IL-1ß and TNF-α in rats treated with Mb [168]. In a murine model of skeletal ageing, Mb inhibited skeletal bone loss [164]. This effect was mainly mediated by Mb antioxidant properties [164]. Due to these properties, Mb has demonstrated effectiveness in reducing cellular damage in a variety of animal models of neurodegenerative and metabolic disorders [169,170,171].

DMSO at low doses acts as a powerful free radical scavenger and also increases the activity of antioxidant enzyme systems [172,173]. DMSO inhibits the activation of NLRP3 inflammasome and suppresses the release of proinflammatory cytokines [174]. Due to DMSO’s antioxidant and anti-inflammatory properties, DMSO has demonstrated neuroprotective effects in models of neuroinflammation and ischemia [175]. In a rat model of traumatic brain injury, administration of DMSO attenuated oxidant injury and improved cognitive function [172].

**Figure 5:**
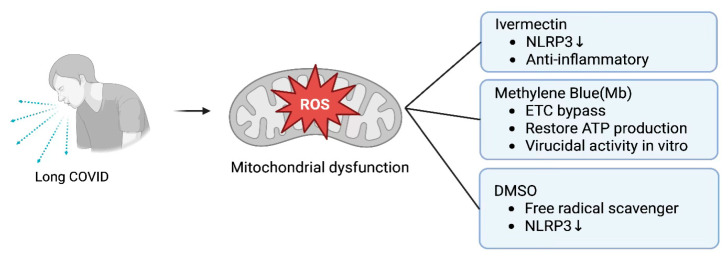
Schematic of mitochondrial dysfunction in long COVID. Excess ROS disrupts the ETC, driving neuroinflammation (brain fog, dysautonomia). IVM suppresses NLRP3, Mb bypasses complexes I–III to cyt c, restoring ATP, and DMSO scavenges free radicals while enhancing antioxidant defenses. ROS = reactive oxygen species; ETC = electron transport chain; IVM = ivermectin; Mb = methylene blue; DMSO = dimethyl sulfoxide.

Thus, ivermectin, Mb, and DMSO, via their pleiotropic effects on inflammatory and oxidant pathways, should be considered in the treatment of long COVID in general and symptoms such as brain fog and dysautonomia commonly encountered in this condition. Figure 5.

### 6.9. Mitochondrial Complications from Spike Protein

In post-acute COVID-19 syndrome (PACS), as well as post-acute COVID-19 vaccination syndrome (PACVS), reduced energy levels, post-exertional malaise (PEM), and brain fog are common issues, affecting large percentages of those affected by either condition. Chronic fatigue syndrome affects 45% (95% CI: 34%–57%) of PAC patients and 69% of PACVS patients [176]. PEM affects 55% (95% CI: 38%–71%) of PAC patients, and 71% of PACVS patients experience "exercise intolerance", the closest surveyed condition to PEM.

Mechanistically, it is known that the spike protein, a common element in both COVID-19 infection and vaccination, can interfere with mitochondrial function and decrease energy production [112]. Spike protein administration is used in the development of animal models for COVID-19-induced brain fog [177].

Apart from COVID-19 or COVID-19 vaccination-related symptoms, reductions in mitochondrial function due to genetic factors or exogenous agents can recreate the clinical presentation of fatigue [178,179], PEM, and/or brain fog. 

Brain fog affects 20% (95% CI: 11%–34%) of PACS patients [180] and 63% of PACVS patients [176]. Brain fog is also associated with mitochondrial dysfunction [181] and is inducible in animal models through spike protein administration [177], as well as other agents that affect the mitochondria, including chemotherapy agents [182] and thiamine deficiency [183].

PEM also shows a high association with mitochondrial dysfunction in PACS patients [184], and the fatigue phenotype can be reliably induced by agents damaging mitochondrial function [178]. In short, mitochondrial dysfunction is a primary driver of the chronic sequelae of COVID-19 or COVID-19 vaccination. After infection, mitochondrial energy production is compromised [93,185,186]. Typically, loss of mitochondrial membrane potential is a signal for recycling of the mitochondria via mitophagy [187], but SARS-CoV-2 spike protein appears to inhibit this process via release of reactive oxygen species (ROS) [188], and possible downregulation of TOM20, which is associated with reduced mitophagy [112,188]. This creates a challenge of lower energy production until the issues can be resolved. One therapeutic strategy may be to upregulate the rate of autophagy through fasting or exogenous compounds [189]. Upregulating mitophagy may merely provide temporary relief, as any new mitochondria produced through mitochondrial biogenesis will be exposed to the circulating spike as well. Spike protein is observed in those with PACS [190,191,192,193,194] as well as PACVS [195], though the association has not been observed in some studies and may only be present for a subset of those with PACS [196].

As such, several therapeutic modalities for PACS and PACVS attempt to limit the damage induced by the spike protein and increase its degradation [189,197,198,199,200]. Besides autophagy [189], spike protein degradation may be induced through proteolytic enzymes [197], including nattokinase [201,202], which has been observed to degrade spike protein [201] and even dissolve microclots [202].

## 7. Conclusions

Mitochondrial dysfunction and oxidative stress are central to the pathophysiology of long COVID and potentially relevant in select post-vaccine syndromes. Ultimately, long COVID is increasingly recognized as a mitochondrial disorder rooted in redox imbalance. By connecting early viral hijack of mitochondrial function to persistent immune dysregulation and metabolic inefficiency, we gain a more precise map of the disease process and, with that, a more straightforward path forward. Translational biomarkers, including MDA, mtDNA, glutathione ratios, and MRS-based indices, offer clinical utility for diagnosis, risk stratification, and therapeutic monitoring. Interventions aimed at restoring redox balance, whether through antioxidant supplementation, metabolic modulators, or tailored nutrition, are promising but require further validation in prospective studies. As the evidence grows, so does the urgency to act. For millions of long-haulers, restoring mitochondrial health may be the key to restoring life itself. Incorporating these biomarkers into clinical practice may enable more targeted and effective management of long COVID in the near future.

## Figures and Tables

**Table 2:** Accessory SARS-CoV-2 proteins impacting mitochondrial function.

Protein	Mitochondrial Target	Function/Key effects
ORF3a	Outer mitochondrial membrane pore-forming subunit ATP/potassium channel; ER–mitochondria contact site	Increase ROS, apoptosis, and decrease INF signaling; disrupts mitochondrial membrane [85,90]
ORF5	MAVS signaling axis	Downregulates MAVS; limits TBK1/IRF3 recruitment; downregulates interferon response [73]
ORF6	SAM complex, outer mitochondrial membrane	Metabolic reprogramming (lipolysis, fatty acid oxidation) attenuates MAVS. Immune suppression and altered mitochondrial proteome [91,92]
ORF9b	TOM70 (outer membrane), RIG-I/MDA5–MAVS	Immune evasion, inflammation, oxidative damage, and altered OXPHOS; inhibits MAVS; decreases interferon I/III signaling [93,94]
ORF9c	Complex I accessory complex (NDUFB9, NDUFAF1), Cristea	Impairs Complex I; increases ROS; induces mitochondrial fragmentation; decrease IFN production; immune evasion, inflammation, oxidative damage, and altered OXPHOS [90,94]
ORF3c	TOM70, TOM20, MAVS modulation (outer membrane)	Alters metabolism; increases ROS; blocks autophagy; increased ROS altered autophagy; Impairs INF and immunosuppression [93,95]
ORF7b	MAVS (outer membrane), MAM (endoplasmic reticulum)	Inhibits MAVS–TRAF6 interaction; increases ROS via interaction with MAM; decrease production of IFN-ß [96,97,98]
ORF10	NIX (outer mitochondrial membrane)	Triggers mitophagy; disrupts MAVS, disrupts mitochondria, and decreases IFN signaling [73,99]
NSP4	BAX (outer mitochondrial membrane)	Induces macropore formation; release of mtDNA, pro-apoptotic effects, and inflammation [100]
NSP8	Colocalizes with the outer mitochondrial membrane	Induces incomplete mitophagy; causes mitochondrial damage; disrupts autophagy; reduces IFN signaling, and dampens innate immunity [101,102]
M protein	MAVS (outer mitochondrial membrane)	Triggers mitophagy; inhibits MAVS signaling, suppresses interferon I and III production, and causes irreversible loss of mitochondrial membrane potential, leakage of cytochrome C, and apoptosis [103,104]
Nucleocapsid	Localizes to the mitochondria and impairs mitochondrial transcription machinery	Increases ROS, decreases, and can also increase ATP production, and inhibits antioxidant enzymes; it increases oxidative stress and, indirectly, causes mitochondrial dysfunction [105,106]
E protein	ER/ERGIC/Golgi membranes; indirectly impacts mitochondria	Disrupts ER calcium stores; impairs ER–mitochondrial Ca^2+^ transfer; contributes to mitochondrial dysfunction [73]

**Table 3:** Long COVID Treatments Targeting Oxidative Stress and Bioenergetics.

Study Type	Study Name	Intervention Specifics	Outcome	Reference
Observational	Coenzyme Q10 + Alpha Lipoic Acid for Chronic COVID Syndrome	500 mg/day CoQ10 + alpha lipoic acid (Requpero®) vs. no treatment	53.5% achieved full fatigue response vs. 3.5% in control; significant symptom reduction	[143]
Randomized cross-over trial	High-Dose Coenzyme Q10 for Post-COVID Condition	500 mg/day CoQ10 for 6 weeks vs. placebo (2×2 cross-over design)	No significant benefit over placebo in reducing post-COVID-19 condition symptoms; both groups improved similarly over time, suggesting natural recovery or placebo effect rather than a treatment effect	[130]
Observational	L-Arginine and Vitamin C for Long COVID	Includes a combination of L-arginine and Vitamin C supplements	L-arginine and Vitamin C group experienced less severe long COVID symptoms, with favorable effects on all symptoms	[144]
Double blind randomized controlled trial	Favorable Antiviral Effect of Metformin on SARS-CoV-2 Viral Load in a Randomized, Placebo-Controlled Clinical Trial of COVID-19	Metformin, fluvoxamine, and ivermectin	Metformin significantly reduced SARS-CoV-2 viral load	[145]
Randomized controlled trial	Vitamins K2 and D3 Improve Long COVID, Fungal Translocation, and Inflammation: Randomized Controlled Trial	Vitamins K2 and D3	Improved number of Long COVID symptoms, significantly lowered markers of inflammation (sTNF-RI, sCD163), oxidative stress (oxidized LDL), and fungal translocation (β-D-glucan)	[146]
One-arm open-label study	The results of a unique dietary supplement (nutraceutical formulation) used to treat the symptoms of long-haul COVID	β-caryophyllene and pregnenolone supplement	Statistically significant improvements in their overall symptoms after 2 and 4 weeks of treatment There were some symptoms, such as fatigue and brain fog, that appeared to respond more than others; however, no baseline presentations were able to predict individual symptomatic responses	[147]
Randomized controlled trial	Eight-Week Creatine-Glucose Supplementation Alleviates Clinical Features of Long COVID	Creatine, creatine, and glucose combined	Significantly elevated brain creatine levels and significantly reduced symptoms such as body aches, concentration difficulties, and headache compared with placebo	[148]
Double-blind, placebo-controlled randomized trial	Potential anti-inflammatory and anti-fatigue effects of an oral food supplement in long COVID patients	*Echinacea angustifolia*, rosehip, propolis, royal jelly, and zinc	Significant reduction in the inflammatory parameters during the OFS period, in comparison to the placebo. Statistically significant increase in serum values of vitamin D after the OFS	[149]
Randomized placebo-controlled trial	Effects of an 8-week high-dose vitamin D supplementation on fatigue and neuropsychiatric manifestations in post-COVID-19 syndrome: A randomized controlled trial	Vitamin D	Improved fatigue, reduced anxiety, and enhanced cognitive function (ACE score: +2.1, p = 0.012), with no meaningful changes in sleep quality, depression, or inflammatory markers (IL-6, CRP), and no serious adverse events	[150]

## Data Availability

No new data were created or analyzed in this study.
